# Urbanization pressures alter tree rhizosphere microbiomes

**DOI:** 10.1038/s41598-021-88839-8

**Published:** 2021-05-03

**Authors:** Carl L. Rosier, Shawn W. Polson, Vincent D’Amico, Jinjun Kan, Tara L. E. Trammell

**Affiliations:** 1 Department of Plant and Soil Sciences, University of Delaware, Newark, DE 19716 USA; 2 Center for Bioinformatics and Computational Biology, Delaware Biotechnology Institute, University of Delaware, Newark, DE 19713 USA; 3 Department of Computer and Information Sciences, University of Delaware, Newark, DE 19716 USA; 4 US Forest Service, Northern Research Station, Department of Entomology and Wildlife Ecology, University of Delaware, Newark, DE 19716 USA; 5 Department of Microbiology, Stroud Water Research Center, Avondale, PA 19311 USA

**Keywords:** Microbial communities, Environmental microbiology

## Abstract

The soil microbial community (SMC) provides critical ecosystem services including organic matter decomposition, soil structural formation, and nutrient cycling. Studies suggest plants, specifically trees, act as soil keystone species controlling SMC structure via multiple mechanisms (e.g., litter chemistry, root exudates, and canopy alteration of precipitation). Tree influence on SMC is shaped by local/regional climate effects on forested environments and the connection of forests to surrounding landscapes (e.g., urbanization). Urban soils offer an ideal analog to assess the influence of environmental conditions versus plant species-specific controls on SMC. We used next generation high throughput sequencing to characterize the SMC of specific tree species (*Fagus grandifolia* [beech] vs *Liriodendron tulipifera* [yellow poplar]) across an urban–rural gradient. Results indicate SMC dissimilarity within rural forests suggests the SMC is unique to individual tree species. However, greater urbanization pressure increased SMC similarity between tree species. Relative abundance, species richness, and evenness suggest that increases in similarity within urban forests is not the result of biodiversity loss, but rather due to greater overlap of shared taxa. Evaluation of soil chemistry across the rural–urban gradient indicate pH, Ca^+^, and organic matter are largely responsible for driving relative abundance of specific SMC members.

## Introduction

Soil microbial community (SMC) plays an essential role in many soil ecosystem functions including litter decomposition, soil-C mineralization, nutrient cycling, and soil structure stabilization/formation. Due to the vital importance of a diverse SMC, several studies have investigated the influence of plants in shaping microbial communities^1^. A broad understanding has emerged that plant traits alter soil chemistry thereby directly or indirectly effecting SMC composition. Tree leaf litter quality/quantity^2^, tree nutrient requirements^3^ and canopy modification of precipitation^4^ have been suggested as drivers of SMC diversity via changes in the soil chemical environment. Additionally, the combination of climatic conditions such as decreased soil moisture^5^ and elevated CO_2_ and/or temperature^6,7^ have also been suggested as influential mechanisms effecting SMC composition. Since trees are directly influenced by climatic conditions, it is possible that the combination of several biophysical and environmental factors influence the structure and function of the SMC. However, few studies have assessed the relative importance of complex interactions occurring between soil physiochemical characteristics, climatic conditions, and tree species on SMC composition.

Trees alter forest climate and modify the flow of many resources to above and below-ground forest organisms^1,8^. Tree species in temperate and boreal forests are considered keystone species^9^, and strongly influence nutrient resources within the soil environment^10^. The influence of tree species on soil chemical environment is driven via several mechanisms including: litter quantity/quality^11^, rate of nutrient and water cycling^12–14^, root exudation^15^, and capacity to acidify soils^16^. Previous research provides evidence that tree alteration of the soil chemical environment can significantly alter the soil microbial community structure via shifts in soil pH^17^, litter quality inputs^18^, and soil carbon and nitrogen concentrations^19^. In addition, anthropogenic activities can also drive changes in forest climate (i.e., increase air temperature coupled with decrease in soil moisture^20,21^) and chemical and nutrient deposition to forests (e.g., heavy metals and nitrogen^22^), which can shift soil properties and nutrient pools ultimately overriding tree influences on the soil environment. We suggest that alterations to the forest climate could supersede tree-driven structuring of soil biochemistry and ultimately SMC.

Forest location within a complex and changing landscape can result in forested ecosystems that are more immediately susceptible to the influence of anthropogenic climate shifts (i.e., higher temperatures), particularly smaller forests embedded within developed landscapes^23^. Watts indicated that 75% of forests within the UK are less than 2 ha^24^. The mid-Atlantic region of the US is largely characterized by small (< 50 ha) temperate-deciduous forests accounting for approximately 30% of total forest area (D’ Amico III et al., *unpublished data)*. Because of smaller forest size coupled with extensive distribution across multifaceted landscapes, small forests are greatly influenced by their proximity to several non-forest environments (i.e., agriculture and urban development) via edge effects^25^. According to Young and Mitchell^26^, forests of 9–10 ha are dominated by edge effects and forests < 1.0 ha are composed entirely of edge habitat. Consequently, forest edges differ significantly from forest interiors due to increased solar radiation and wind penetration, which in turn lowers soil and litter moisture content^27–29^ and potentially alters both soil carbon and nutrient cycling. However, despite the understanding of edge effects on key soil processes few studies have investigated these impacts on soil microbial communities^29,30^ and we know of no research investigating the effects of urban forest edges on SMC structure and distribution.

Urban forest trees offer the ideal experimental system to evaluate whether natural (i.e., plant) or anthropogenic (i.e., altered environment) factors have the greatest effect on SMC structure and distribution. Knowledge of potential controls governing the ecosystem structure (e.g., community patterns) and function (e.g., nutrient turnover) of urban soils is of critical importance as the transformation of natural and agricultural land towards urban and suburban settlement continues to expand^31^. Urban land development alters numerous environmental factors including air temperature, rainfall quantity, and nutrient deposition; potentially affecting critical soil ecosystem services (i.e., carbon storage, soil moisture holding, and nutrient cycling). Recent research efforts have begun to investigate the intricate connections between SMC composition/diversity/functionality and urbanization. Evidence suggests that microbial composition is influenced within highly urbanized areas such as soils along industrialized roadways^32^. Epp Schmidt et al.^33^ found that urbanization results in biodiversity loss of specific groups of soil organisms, specifically ectomycorrhizae. However, the question remains whether SMC structure/composition responds to abiotic pressures on the plant community. Reese et al.^34^ proposed that both abiotic and biotic drivers influenced by urbanization alter SMC composition but not richness, indicating that ecosystem services provided by the SMC may be resilient to urbanization. However, Wang et al.,^35^ found differences in the nitrifying/denitrifying community structure within urban lawns compared to rural farmland soils suggesting a potential decline in ecosystem functionality. While these studies provide pivotal evidence indicating potential loss of microbial diversity, they do not consider the influence of abiotic pressures (i.e., urbanization) on plants (i.e., biotic factors) shaping the SMC structure/composition.

**Figure 1:**
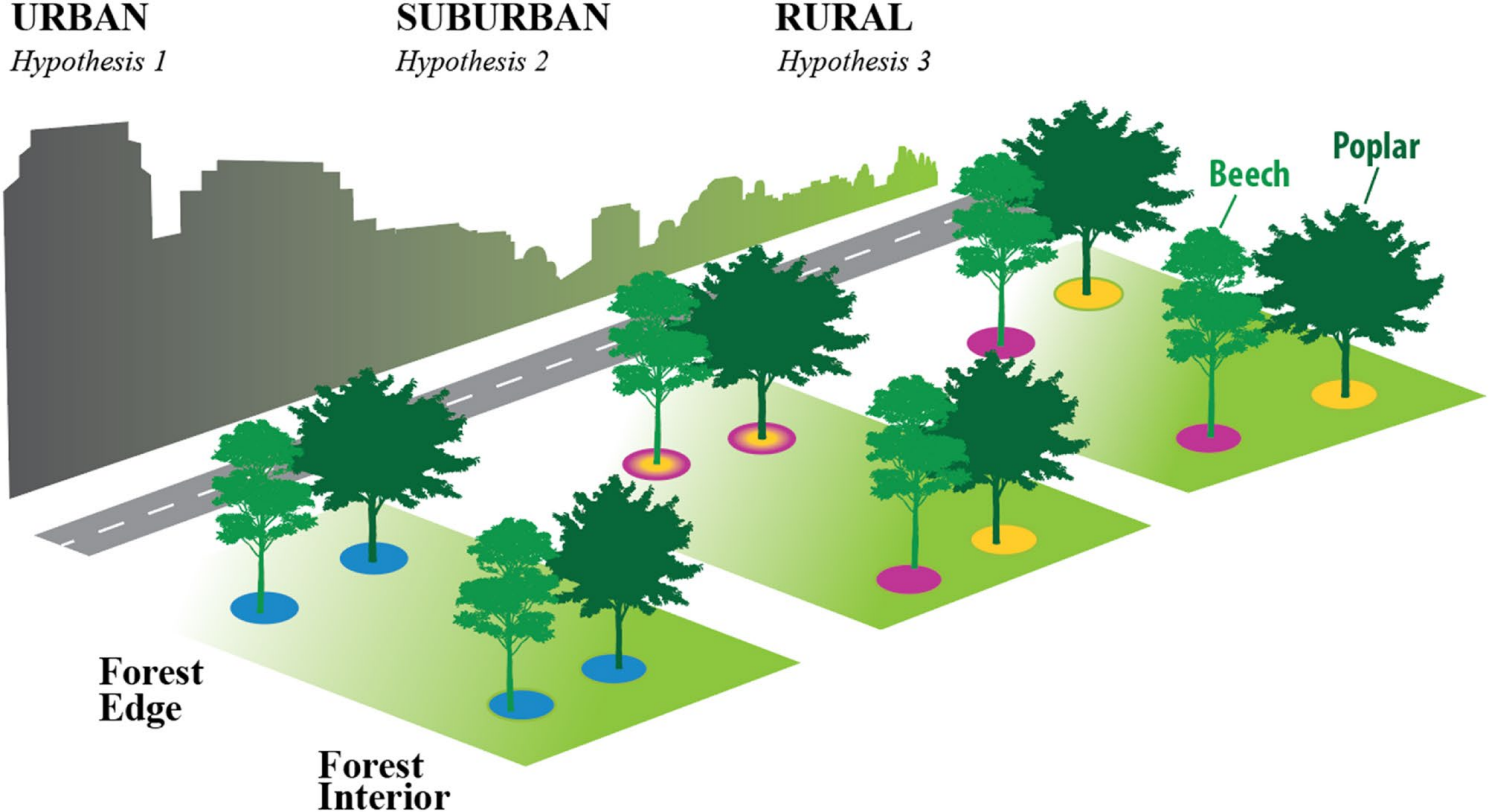
Conceptual diagram of hypothesized soil microbial community (SMC) structural changes [represented at tree bole via colored circles (for example, unique colored circles signify dissimilarity of the SMC)] in response to tree species (beech vs. yellow poplar) forest type (rural, suburban, and urban) and location (interior vs. edge). Additionally, diagram provides on overview of our sampling methods as all forest edge locations were directly adjacent to a roadway and interior locations were ~ 100 m from defined roadway edge. H_1_: Intensified urban pressures at both forest edge and interior will drive complete SMC structure similarity despite of tree species influence. H_2_: SMC structure will begin to converge at forest edge regardless of tree species due to the overriding effect of environmental inputs; however, suburban interior forest SMC will remain dissimilar due to tree overriding environmental effects. H_3_: Dissimilar SMCs will occur as a result of tree influence due to moderate fluctuations of environmental conditions within both edge and interior rural forests.

The overarching goal of this study is to evaluate the response of the SMC composition to tree species (i.e., *Fagus grandifolia*, *Liriodendron tulipifera*), forest location (i.e., edge vs. interior), and forest type [i.e., rural, suburban, urban (Fig. 1)]. Potential links between the structure and diversity of the microbial community and changes in edaphic conditions resulting from increased proximity to urbanization were also analyzed. The objectives of this study were: (i) to determine if tree species has a greater influence on bacterial community composition than forest edge effects (e.g., edge vs. interior) and/or urbanization pressure., (ii) to identify bacterial groups driving community composition differences (i.e., similarity vs. dissimilarity) corresponding to tree location and forest type, and (iii) to categorize urbanization pressures as determined via soil chemistry observations (i.e., pH, organic matter, heavy metals and C/N) in order to identify factors altering soil rhizosphere bacterial community structure of urban soils. We hypothesized that bacterial community composition would largely be governed by tree species; however, we expected forest location and type to override tree effects resulting in a loss of species diversity as well as convergence of the microbial community particularly within urban forests. Specifically, we expected significant differences in edaphic conditions resulting from forest location (i.e., interior vs. edge) and urbanization pressure to drive SMC composition as well as distribution.

## Results

**Figure 2:**
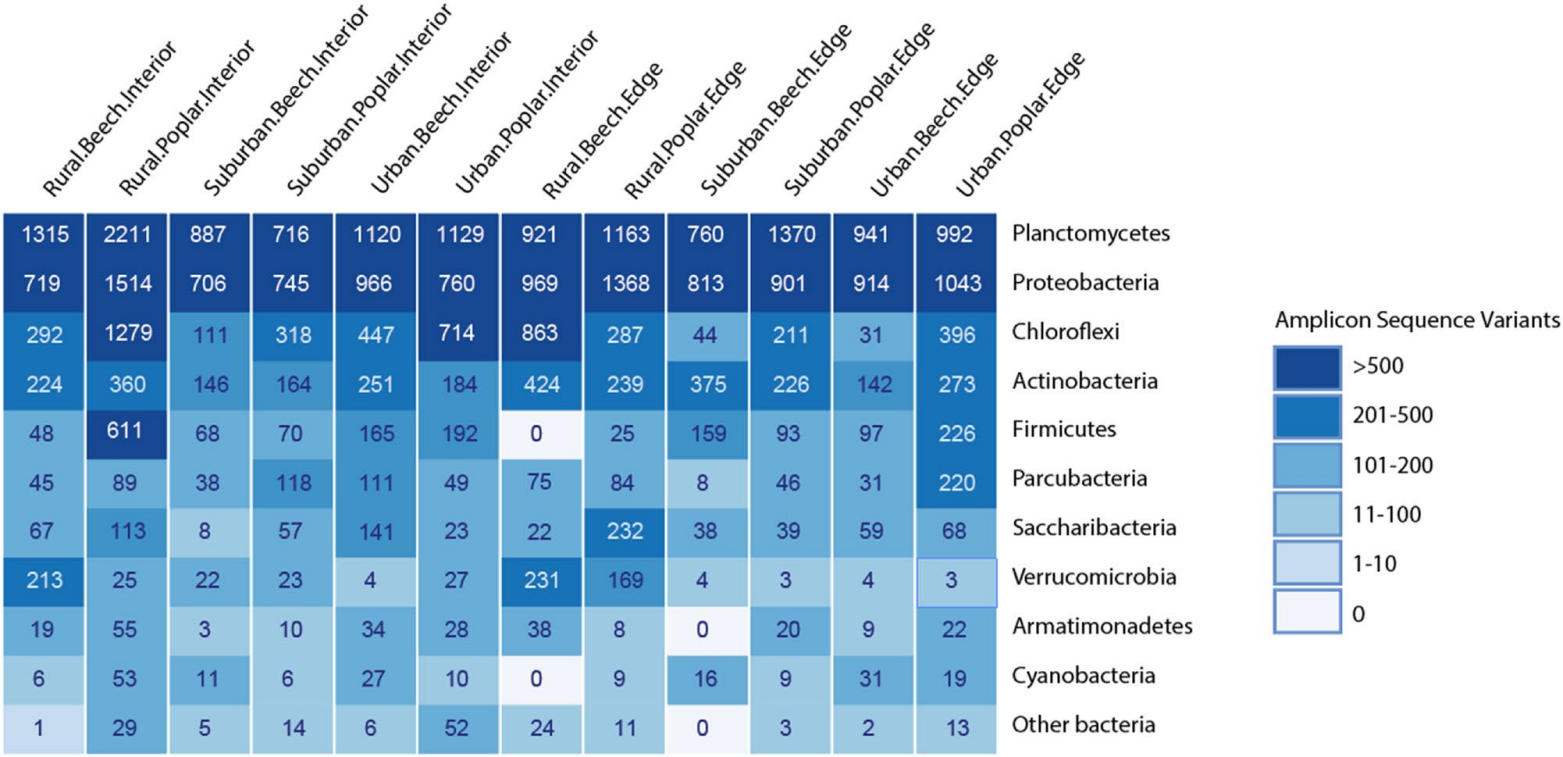
Heatmap depicting average relative abundance of the soil microbial community at the phyla level. Numbers within boxes indicate ASVs identified for each tree, forest, and location.

**Figure 3:**
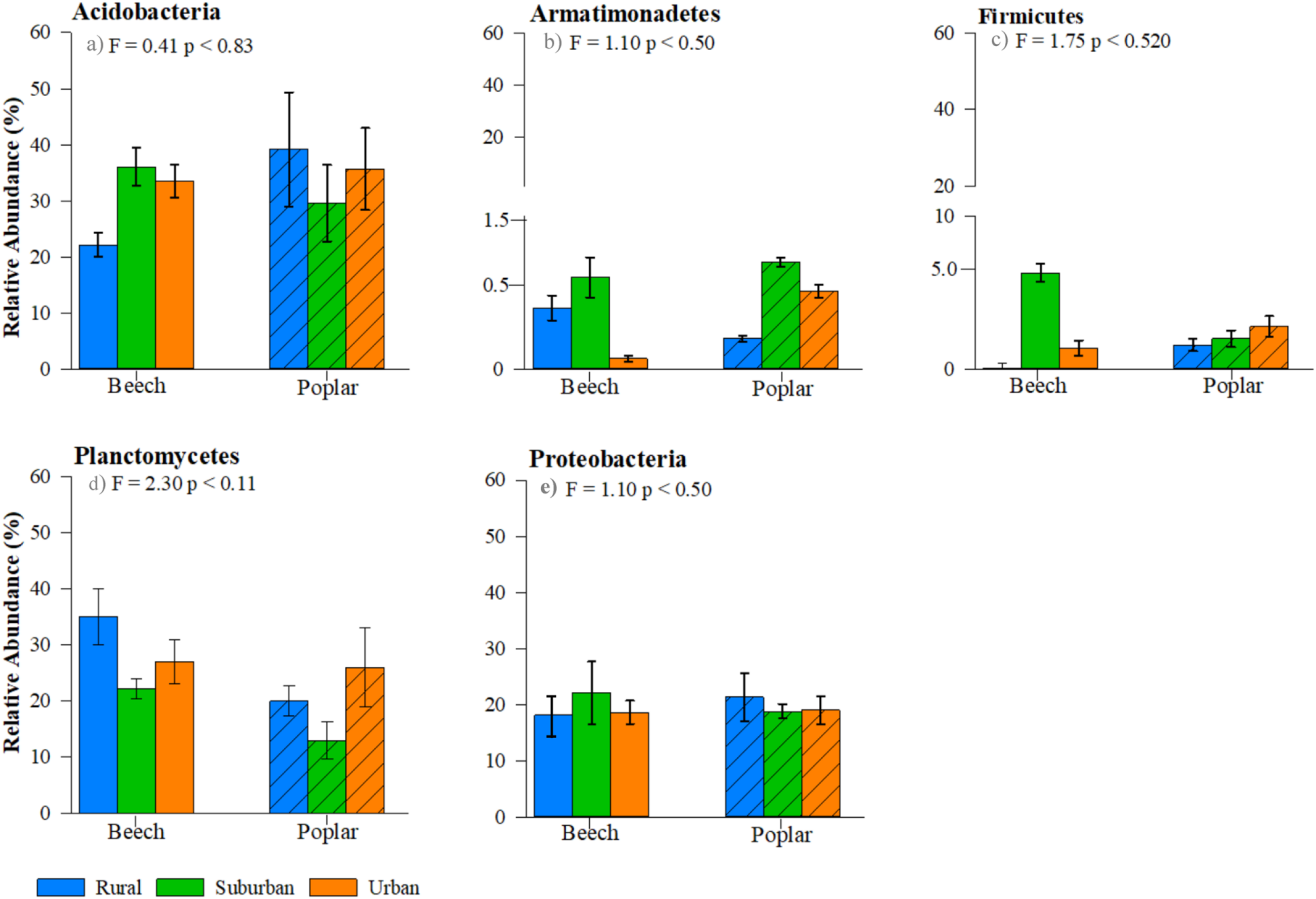
Relative abundance (%) of the dominant 16S rRNA gene sequences isolated from microbial community members at the phylum level (mean ± standard error) significantly influencing observed differences in NMDS plots within forest interiors. Samples are separated by forest type: urban (orange) suburban (green) and rural (blue) and tree (column pattern).

**Figure 4:**
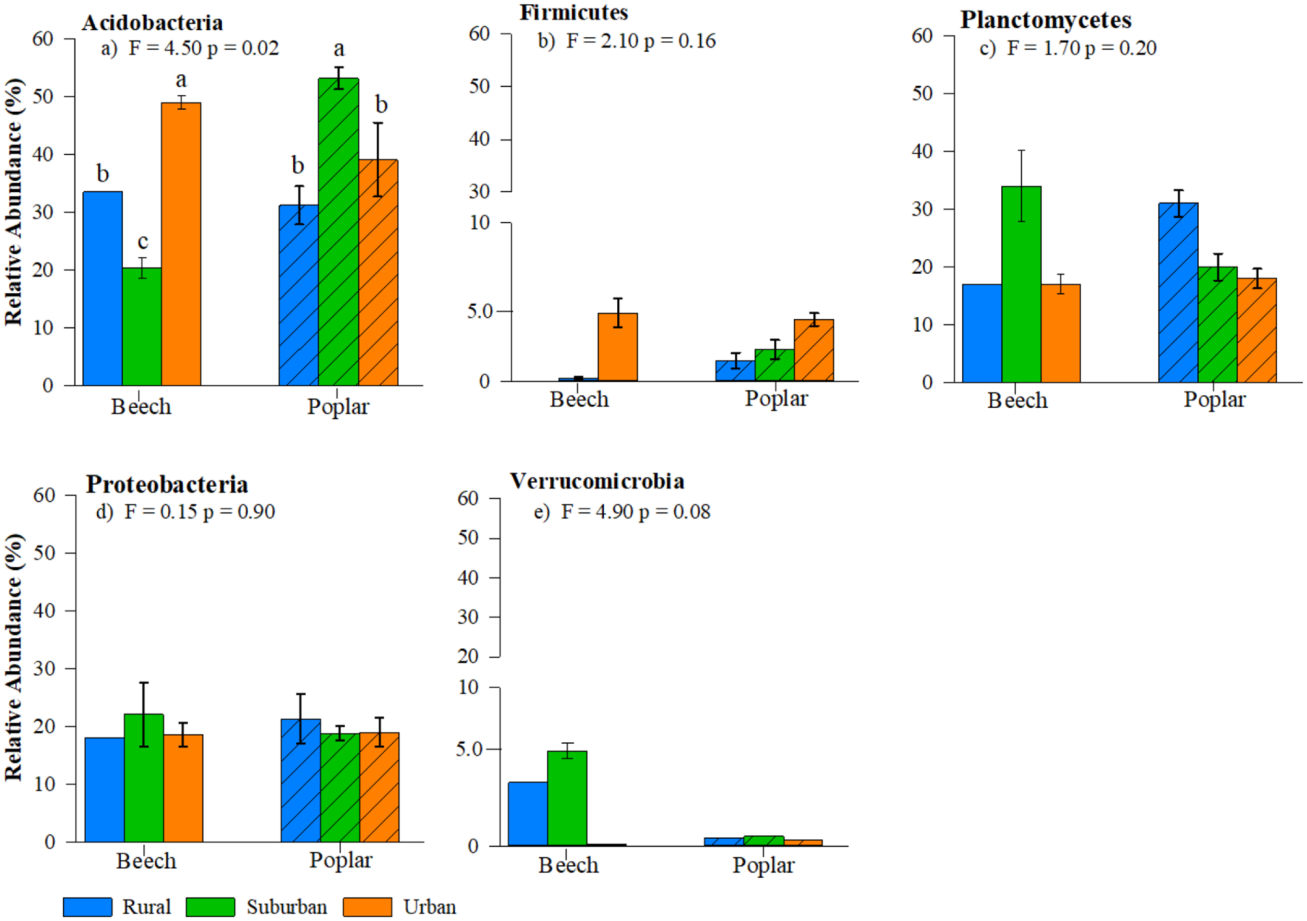
Relative abundance (%) of the dominate16S rRNA gene sequences isolated from microbial community members at the phylum level (mean + standard error) significantly influencing observed differences in NMDS plots along forest edges. Samples are separated by forest type: urban (orange) suburban (green) and rural (blue) and tree (column pattern). Significant differences are depicted when letters above the bars are different (*p* < 0.05).

Twelve dominant bacteria phyla (> 0.1% relative abundance) were identified across all forest types, location, and tree species (Fig. 2). The most abundant phyla (in descending order) were Planctomycetes, Proteobacteria, Chloroflexi, and Acidobacteria comprising approximately 77% of identified bacterial communities (Fig. 2). Relative abundance (%) of predominant phyla (> 0.5%) for interior forest microbial community composition indicates that the communities did not significantly differ across forest type or tree species (Fig. 3). A similar trend was found for major groups within edge dominant microbial communities (Fig. 4); however, we did measure significantly lower abundance of Acidobacteria (p < 0.02) and a potential trend in Verrucomicrobia (p < 0.08) in beech rural forest edges (Fig. 4a,e). This observation is further supported by species evenness (Fig. Sup 1a,b) and richness (Fig. Sup 1c,d); no significant differences were measured between forest type, forest location, or tree species. However, a possible trend in greater species evenness was measured in beech trees in the interior of rural forests compared to the interior of urban and suburban forests (Fig. Sup 1a, p < 0.08). Additionally, we observed a similar trend in greater species evenness for beech rural and suburban edge trees compared to urban edge trees Fig. Sup 1b, p < 0.10), and greater species richness in rural and urban compared to suburban beech trees along the forest edge (Fig. Sup 1d, p < 0.07).

**Figure 5:**
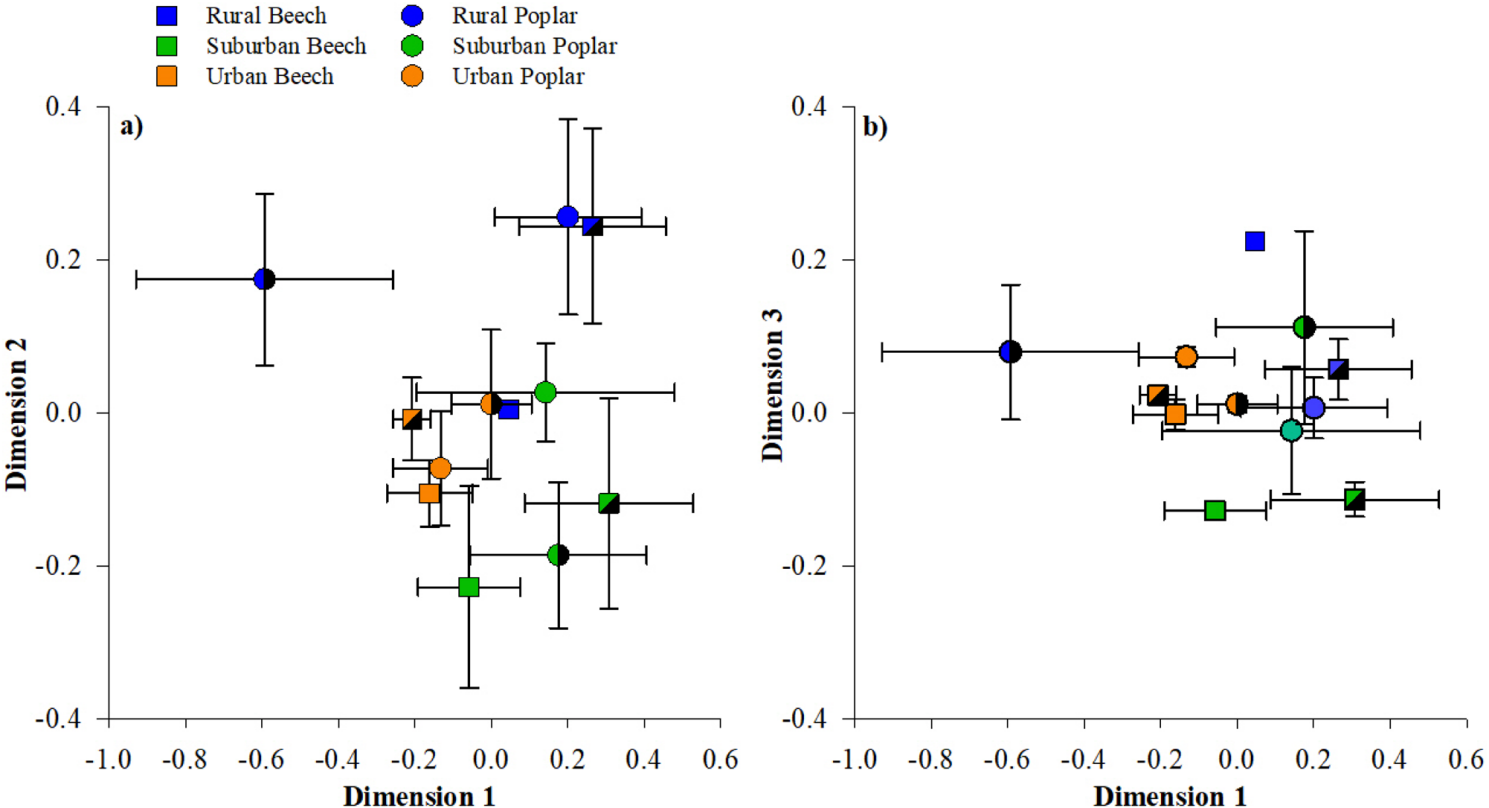
Nonmetric multidimensional scaling (NMDS) ordination comparing beech and yellow poplar microbial community (phyla level composition) across 3-forests (rural-suburban-urban; and 2-locations (Interior: open symbols vs. Edge: hashed symbols) across a) Dimension 1 and Dimension 2 and b) Dimension 1 and Dimension 3. Samples are color-coded to indicate the different forest types. Each point represents the average of three sampled trees (± standard error).

To determine the overall influence of forest type, forest location, and tree species on SMC structure, we conducted community analysis using 25-distinct phyla resulting from our sequencing efforts. NMDS analysis identified 6-phyla within interior and edge samples responsible for driving observed patterns in SMC structure. Relative abundance of 6 phyla accounted for 84% (± 2.2) of all sequences in beech and 83% (± 2.8) in yellow poplar interior sites. However, three bacterial phyla influenced the NMDS loadings of both interior and edge sites (Fig. 5). Acidobacteria was negatively related to dimension 1, and Verrucomicrobia and Planctomycetes were positively related to dimension 2 (Fig. 5). In the interior forest sites, the greatest separation in the SMC was between rural forest beech trees compared to suburban and urban beech trees along dimension 1 (Fig. 5a), where Acidobacteria was least abundant under beech trees in rural forests (Fig. 3a). Along dimension 2, we observed separation of the interior microbial community as a function of forest type (Fig. 5a), whereas dimension 3 provided limited separation (Fig. 5b). Along dimension 2, rural beech and poplar trees separated from urban and suburban trees due to more abundant Planctomycetes in the interior of these forests (Figs. 3d, 5). Rural beech trees and suburban beech and poplar trees showed greater dispersion (i.e., variation in species composition) around the centroid compared urban forest trees (Fig. 5a).

At the forest edge, there is limited separation in the microbial community along dimension 1; however, we observed greater separation between the rural and urban/suburban forests along dimension 2 (p < 0.02; Fig. 5a). This pattern was largely driven by the microbial community composition in both the Plantomycetes and Verrucomicrobia phyla. Edge poplar trees in rural forests had greater abundance of Planctomycetes than in urban forests (Fig. 4c), whereas beech trees in rural and suburban forests had greater abundance of Verrucomicrobia than in urban forests (Fig. 4e). Along dimension 3, we observed separation in the beech rural microbial community compared to the urban and suburban forest sites, which was influenced by greater abundance of Parcubacteria and Chloroflexi phyla (positive loadings on dimension 3; Fig. 5b). Finally, the greatest variation around the centroid occurred in rural and suburban poplar trees along dimension 1 (Fig. 5a).

NMDS analysis of interior forest soils suggests that 6-phyla are responsible for driving observed patterns of the SMC structure across tree species, and forest type (Fig. 5a,b and Fig Sup 2a-d), using this understanding we assessed 210-genera within the 6-phyla of interest in an effort to identify specific bacteria groups influencing NMDS loadings. A detailed analysis of the relative distribution of interior soil bacterial community at genus level revealed significant differences in the Proteobacteria, Firmicutes, Aramtimonadetes, and Planctomysetes phyla across both trees and forest type within forest interiors accounting for 9 specific bacteria genera of 210 analyzed (Sup. Table 1). Specifically, we observed a significant reduction in *Bradyrhizobium, Chthonomonadaceae* and *Tepidisphaerale* as beech soils were exposed to increasing urbanization yet, yellow poplar soils remained unaffected (Sup. Table 1). Additionally, Halanaerobiales (Firmicutes) increased in relative abundance within beech soils as urbanization increased, however, was significantly diminished in urban yellow poplar soils compared to rural (Sup. Table 1). At the forest edge, we observed significant changes in the relative abundance of 15 bacterial genera (Sup. Table 2) of 220 genera potentially influencing NMDS loadings (Fig. 5a,b and Fig Sup 2a-d), this in 3 × greater than changes measured within forest interior [i.e., 5 bacterial genera (Sup. Table 1)].

**Figure 6:**
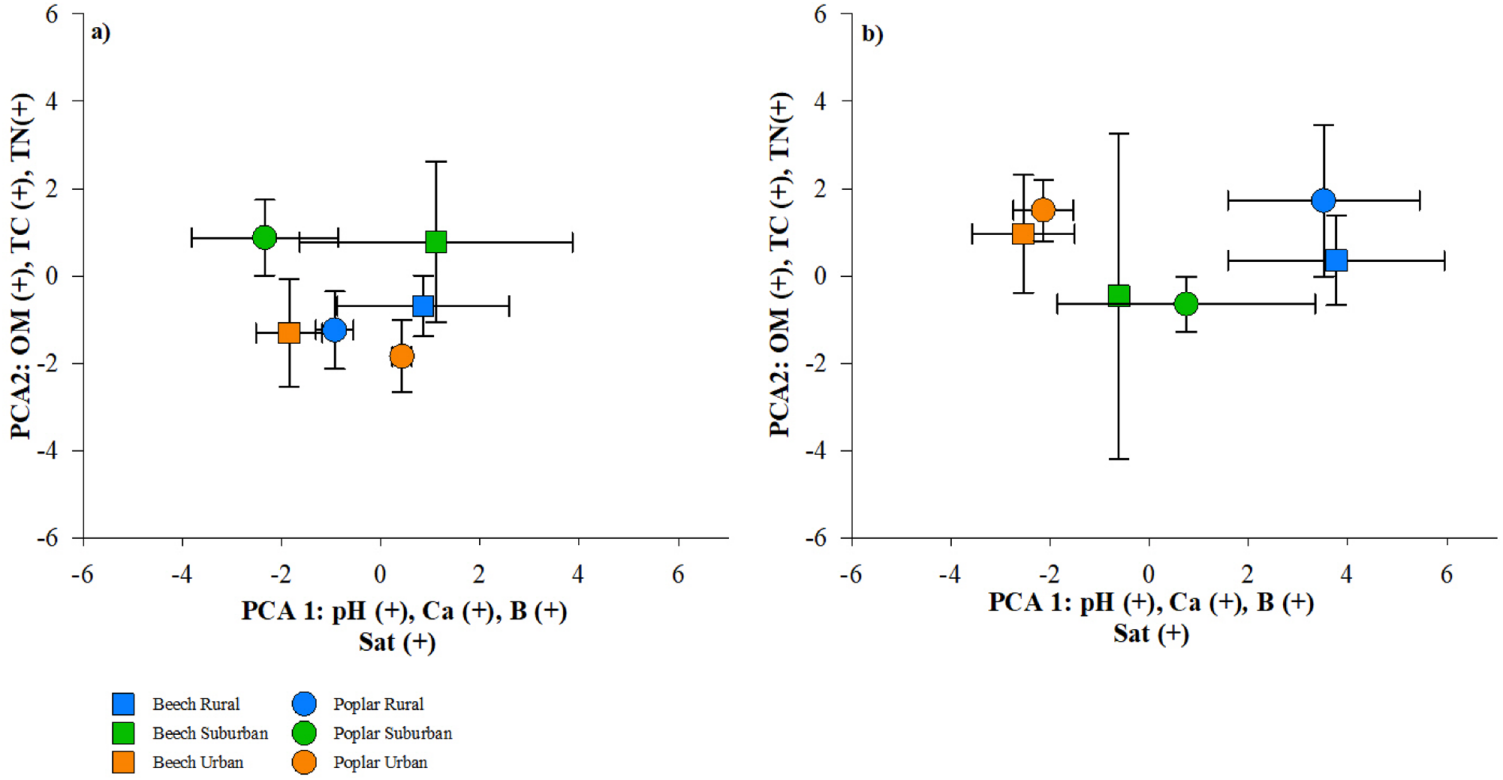
Principal Component Analysis (PCA) plotting PC1 and PC2 for soil samples collected from forest interior **(a)** and forest edge **(b)** comparing beech and yellow poplar (difference by shape) across rural–urban gradient (distinguished by color). Each point represents three sampled trees (± standard error).

**Figure 7:**
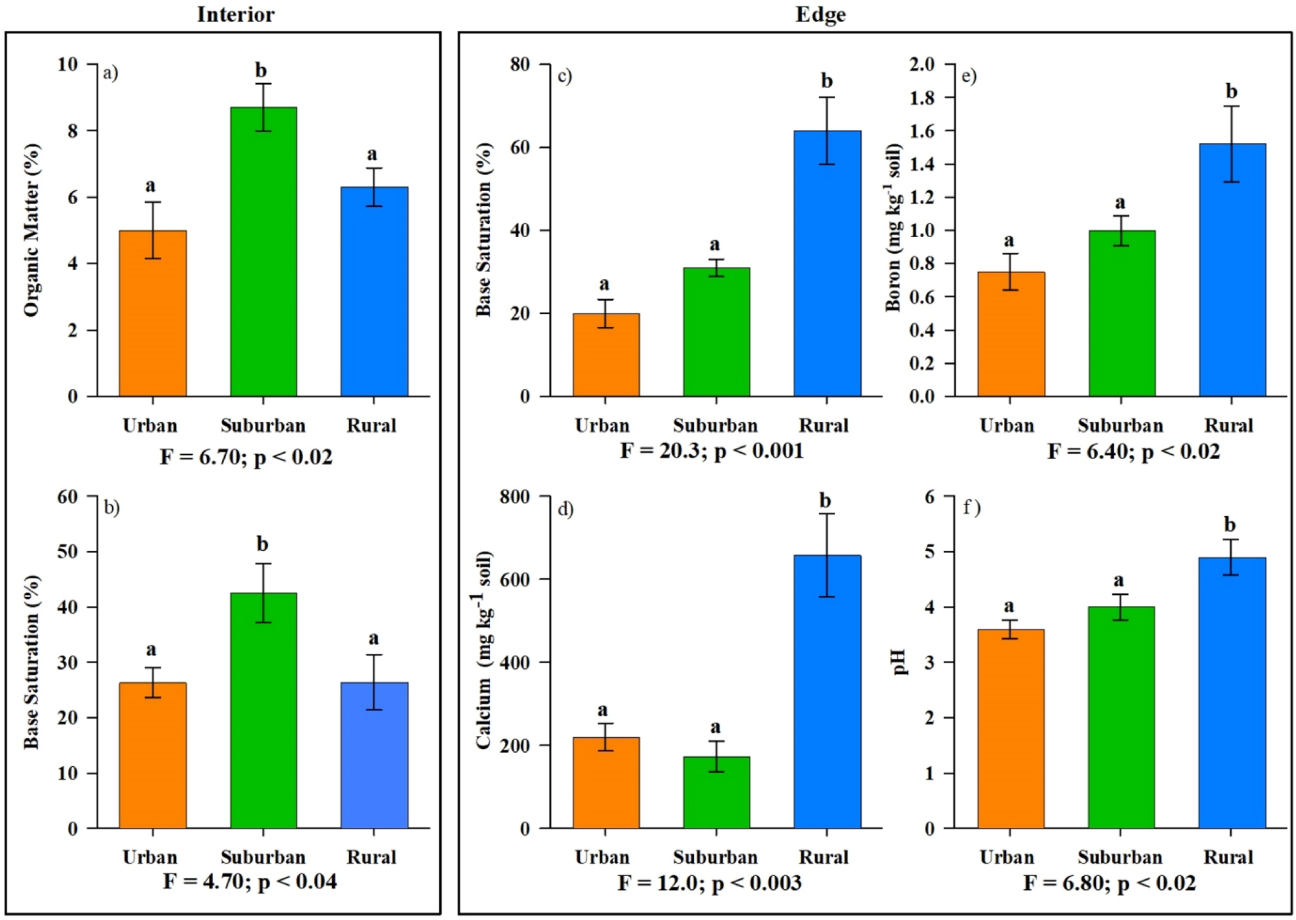
Significant soil chemical properties as defined by PC1 and PC2 in urban (orange) suburban (green) and rural (blue) forests for both interior and edge soils. Bars represent the mean (± 1 SE) of beech and yellow poplar soils combined (n = 6). Significant differences are denoted when letters above the bar (*p* < 0.05).

Soil chemistry characteristics were analyzed using PCA in order to assess differences in forest type and location, and the first two principal components of interior soils accounted for 37% and 20% of the total variance (Fig. 6a). The variables most strongly loaded on principle component 1 were pH, Ca, B, and base saturation with significant positive loadings (Fig. 6a). Along principle component 2, soil OM, TC, and TN were the most significant postive loadings (Fig. 6a,b). Both beech and yellow poplar trees in the interior separated along PC1; however, the position of urban beech and yellow poplar trees were reversed when compared to suburban and rural trees (Fig. 6a). In the interior, beech trees in urban forests had lower pH, Ca, B, and base saturation compared to suburban and rural forests, whereas yellow poplar trees in urban forests had greater pH, Ca, B, and base saturation than suburban and rural forests (Fig. 6a). Specifically, beech rural and suburban soils were significantly less acidic (pH  4.3, 4.4, respectively) and contained greater concentrations of Ca (360, 810 mg kg^1^, respectively) compared to urban soils (pH 3.7 , Ca = 150 mg kg^1^; Sup Table 3). In the forest interior, surburban soils for both tree species separated from rural and urban soils along PC 2 (Fig. 6a), and this was influenced by significant differences in OM (Fig. 7a. p < 0.02). However, no significant differences were observed in TC or TN for any trees regardless of forest type in the interior (Sup Table 3). Along the forest edges, we observed the same pattern across tree species where urban and suburban soils have lower pH, Ca, B, and base saturation than rural forests (Figs. 6b, 7c–f). Alternatively, urban, suburban, and rural forest edge soils had similar OM, TC, and TN (PC2, Fig. 6b; Sup Table 4).

Across both tree species, interior forest soils differed in OM (%) and base saturation (%) throughout the urban–rural gradient. Interior suburban trees had significantly greater OM and base saturation than either urban or rural trees (Fig. 7a,b). Additionally, plant available nutrients differed across forest type for Fe (F = 9.80, p < 0.01, significantly elevated within beech urban soils) and P (F = 6.80, p < 0.02, significantly diminished in suburban beech soils) in interior soils (Sup Table 3). Finally, we measured significant differences in K concentration across the rural–urban gradient for both beech (greater under rural) and yellow poplar (elevated under urban) trees in edge locations (Sup Table 4).

## Discussion

Soil microbial community (SMC) composition was more similar in the interior of our suburban and rural forests than in the urban forests supporting our hypothesis that SMC converges with more urbanization surrounding the forests. Several environmental conditions, such as those found within urban environments (e.g., extreme temperature and drought), can act as a filter leading to community convergence (i.e., similarity) by favoring tolerant species^36,37^. In contrast, divergence in community composition is expected in productive systems, like our rural forests, leading to greater competition and community structure dissimilarity^38^. Fluctuations in both environmental conditions and tree species (e.g., tree productivity) support the coexistence of a biologically diverse and stable SMC^39^.

The relative abundance of dominate phyla is a measure of interconnections between trees species, location, and proximity to urbanization on SMC structure. Across our forests, we found modest differences in dominant phyla between urban, suburban, and forests suggesting that urbanization is not a filter reducing SMC biodiversity^32,33,40^. The capacity of the SMC to resist selective stressors is tightly linked to a high degree of species richness and evenness. Environmental stress endangers microbial communities lacking functional redundancy^41^. Initial community structure before a pulse stress or disturbance has a large influence on SMC recovery^42^. While microbial communities may decline immediately following an extreme environmental stress, SMC return to a consistent community structure once the disturbance subsides suggests that soils have an internal robustness to extreme disturbances^37^.

Across our urban, suburban, and rural forest interiors, species evenness and richness remained stable in yellow poplar soils (Fig. Sup 1a,c). Alternatively, we found a trend for declining species evenness and increasing species richness in suburban and urban beech soils compared to rural soils suggesting soils underneath beech trees demonstrate greater sensitivity to urbanization pressures than underneath poplar trees. In urban forests, we found a significant reduction in the relative abundance of free-living *Bradyrhizobium* community in soils underneath beech trees and decreases in *Bradyrhizobium* abundance have been found to be correlated with soil-N content and pH^43^. In our study, soil pH decreases as urbanization increases, yet soil-N content remains the same suggesting that soil pH may have a greater effect on free-living Bradyrhizobium abundance than total N. The relative abundance (%) of *Bradyrhizobium* had a significant positive relationship with soil pH (r^2^ = 0.40, p < 0.03, Fig Sup 3a), however, only a modest trend was measured for total-N (r^2^ = 0.18, p < 0.18, Fig Sup 3b).

Similarly, we found *Firmicutes Halanaerobiales* increased in relative abundance in beech soils as urbanization increased (Sup Table 1), which are obligatory anaerobic, moderately halophilic bacteria requiring high NaCl concentrations for optimal growth^44^. While representatives of this microbial group are typically isolated from sediments of several hypersaline lakes^45^, we know of no study has identified Halanaerobiales communities within forest soils. However, shifts in the overall soil microbial community structure have been identified within soils due to tree species-specific canopy capture and redistribution of NaCl to the soil environment via throughfall^4,46^. Potentially both urban beech and yellow poplar trees could modify the soil microbial community via canopy capture of various urban associated particulates (i.e., aerosols, nutrients, heavy metals) redistributing them to the soil surface via either stemflow or throughfall. However, the relative abundance (%) of *Firmicutes Halanaerobiales* decreased with increasing soil pH (r^2^ = -0.40, p < 0.03, Fig Sup 4a) and soil Ca concentrations (r^2^ = -0.36, p < 0.04, Fig Sup 4b) indicating soil chemistry constituents beyond NaCl may be influencing *Firmicutes Halanaerobiales* abundance.

Changes in the relative abundance of dominate phyla within a given soil environment often occur under elevated conditions such as saline soils^47^, drainage of flooded soils^48^, differing management practices (conventional vs. organic agriculture^49^) and nitrogen additions^50^. The lack of difference in relative abundance within forest interior soils is not surprising since the majority of soil chemistry parameters measured were similar across tree species, forest edge and interior, and urban, suburban, and rural forests (Sup Table 3). However, soils underneath beech urban and poplar suburban trees contained greater relative abundance of Acidobacteria, which are classified as one of the most abundant ubiquitous bacterial phyla isolated from soil environments^51^. Acidobacteria are often slow growth oligotrophs and their abundance within a soil community is contingent on acidic pH^8^. Soils underneath beech urban and poplar suburban trees had the lowest pH across all forests (Sup. Table 4). Environmental conditions associated with urbanization and tree species-specific influences on soil chemistry resulted in modest differences in SMC composition in the interior of our forests, yet maintained diverse communities compared to the forest edges.

Due to the expansion of anthropogenic activities including development of urban centers and conversion of forests to agricultural usage the creation of small forests and coincidently forest edges are ever increasing (D’Amico et al. *unpublished data*). Forest edges differ significantly from forest interiors with regards to environmental factors such as soil temperature, light availability, and wind speed, which in turn affects soil moisture status^52^. Additionally, forest edges especially those connected to urban centers are prone to deposition of nutrients as well as pollutants^53^. Given the combination of both environmental and chemical factors effecting forest edges it is highly reasonable to expect differences within microbial communities. The greatest divergence in SMC composition occurred along edges in rural and suburban forests, yet urban forests clustered tightly together suggesting SMC convergence (Fig. 5b). The forest edge appears to have a sorting effect influencing certain bacterial taxa in forests with less surrounding urbanization^29,54^.

Members of Verrucomicrobia have been identified within a variety of plant-soil ecosystems, where the majority of studies suggest that greater relative abundance are well correlated with soils containing lower P and K concentrations^55–57^. The beech trees in suburban forest edges maintained greater relative abundance of Verrucomicrobia (Fig. 4e) and had the lowest concentrations of soil P and K (Sup. Table 4). However, across all sites, the relative abundance (%) of Verrucomicrobia had no relationship to P (p < 0.60), K (p < 0.35) or Ca^2+^ (p < 0.94) concentrations. Similarly, SMC separation across tree species and urban, suburban, and rural forests was influenced by the community composition of Planctomycetes and Verrucomicrobia. Planctomycetes represent an extremely diverse microbial community within arable soils^58^. However, despite this abundance, extraordinarily little is understood regarding the environmental factors that shape the community abundance or diversity of Planctomycetes. In soils isolated from an apple orchard, the diversity of Planctomycetes was driven by soil organic matter, Ca^2+^ content, pH, and soil N concentrations^59^. Along forest edges, differences in soil Ca^2+^, pH, organic matter, and total N were responsible for separating urban and suburban forests from rural forests overriding potential tree influences shaping Planctomycetes community patterns. However, across all our sites the relative abundance (%) of Planctomycetes was not influenced by Ca^2^ concentrations, organic matter, or soil pH (p > 0.10).

Soil chemistry associated with our rural–urban gradient was important in determining bacterial genera patterns across forest edges. In both beech and yellow poplar edge soils, we found Proteobacteria Nitrosomonadaceae relative abundance (%) significantly decreased as urbanization increased where soils were more acidic with lower Ca concentrations ( Fig. Sup 6a-b). Significant reductions in the relative abundance of ammonia-oxidizing bacteria were observed as a result of Ca amendment effects on increasing soil pH in mineral soil horizons^60^. We found that as soil pH (p < 0.0001, Fig. Sup 6a) and Ca^2+^ concentrations (p < 0.001, Fig Sup 6b) positively increased as Nitrosomodance relative abundance (%) increased. Similarly, we observed a steady increase in the relative abundance of Acidobacteria Bryobacter within the soils of both beech and yellow poplar trees as urbanization increased. Acidobacteria Bryobacter, characterized as an aerobic chemo-organotrophic bacterium capable of utilizing various sugars, polysaccharides, and organic acids, plays a significant role in the C cycle^61^. In our forest edges, abundance of Bryobacter increased with soil OM content and concentrations of P with urbanization (Sup Table 2 & 4) suggesting Bryobacter is directly linked to P availability and soil OM content^62^. However, we observed no relationship between Bryobacter relative abundance (%) and P concentrations or organic matter across all of our forest soils (p > 0.10).

The soil ecosystem is an immensely complex heterogeneous environment governed by numerous interactions and microbial community structure is directly connected to both chemical and physical processes. In order to identify a crucial process controlling SMC composition/diversity, a myriad of studies have sought to identify core chemical and physical mechanisms shaping the composition of the SMC. It is generally well accepted that soil pH is a predominate driver of SMC composition^63^. Although, several additional core-drivers have been suggested including organic matter content^64^, ratio of C:N:P^65^, salinity^66^ and CO_2_ concentrations^67^. However, recent studies considering environmental factors, such as soil moisture and temperature status, have observed alterations in soil chemistry^68^ influencing rates of organic matter decomposition^69^ which in turn influence SMC biodiversity.

Urbanization alters environmental conditions known to influence SMC composition factors. In cities, chemical (e.g., atmospheric deposition of nutrients and pollutants^70^), climatic (e.g., heat island effects^71^), and physical (land use change^72^) alterations have been linked to changes microbial community diversity^32,35^ potentially altering several soil ecosystem services (i.e., nutrient retention, C sequestration). Across our rural–urban gradient, interior suburban forests soils lie within a unique junction of forest types, where they are buffered from the full impact of urbanization, yet prone to some urban pressures. Suburban forests have significantly greater OM (%) than urban and rural forests in our study. It is plausible the increase in suburban OM is the result of mild heat island effect increasing plant productivity and OM inputs to soil while maintaining intermediate OM decay rates relative to urban and rural soils^73^. The composition of the SMC is fundamentally important in both the chemical and physical development of the soil ecosystem, understanding mechanisms that effect SMC composition is key to predicting and managing ecosystem development^74^.

In conclusion, forest proximity to urbanization increased SMC similarity (i.e., convergence) in both beech and yellow poplar soils suggesting that environmental/climate conditions override tree influences to shape SMC structure. However, in forests connected to less-urban environments (i.e., rural and suburban), as environmental pressures subside tree influence on SMC structure increases. Exploration of environmental/climate effects on soil chemistry difference across our rural–urban gradient suggest that shifts in SMC convergence/divergence maybe linked to urbanization effects on soil pH, Ca^+^ concentrations, and OM content. Future studies should consider identifying the core microbiome, assessing their functions and gene regulation, and measuring dynamic shifts in both soil moisture and temperature across urban–rural forest gradients. Deciphering whether the functionality of the SMC shifts as communities converge in urban forests is important for establishing potential impacts on vital ecosystem processes, including decomposition and nutrient cycling, that could in turn alter future plant community composition and sustainability of urban forests.

## Materials and methods

### Site description

In 2009, researchers at the USDA Forest Service and University of Delaware began a long-term urban forest network, the FRAME (FoRests Among Managed Ecosystems, http://sites.udel.edu/frame/). Forests randomly selected across Coastal Plain and Piedmont hardwood forests were located on public lands for long-term establishment and study. All forest study plots were positioned along an edge of the forest to capture potential edge effects. The FRAME urbanization gradient extends from Newark, DE, which has a population of 31,454 and a mean density of 1,403 people km^2^ (US Census Bureau, 2010) to nearby rural landscapes. We selected three forests that span the urban–rural gradient in the FRAME and varied in impervious surface cover (urban = 36.5%, suburban = 11.3%, rural = 6.1%) and population density (urban = 4457, suburban = 1734, rural = 947). Within each forest, we selected three American beech (*Fagus grandifolia*) and three yellow poplar (*Liriodendron tulipifera*) trees within 10 m of the forest edge and at least 100 m from the forest edge (i.e., forest interior). All trees (n = 36) had similar diameter at breast height [dbh (range = 53.4 ± 2.5)] and all forests had intact canopy since 1937 (i.e., aerial imagery; 74).

### Soil sampling

Thirty-six 1-m × 2.5 cm (diameter) soil cores were collected from 18-beech and 18-yellow poplar trees between June and July 2017 approximately 0.5 m from the base of the tree trunk after removal of the leaf litter layer, and immediately stored on ice. Within 12 h of sampling, each core was subdivided into 4-distinct horizons (i.e., 0–10, 10–30, 30–60, and 60 + cm) and 2.5 g samples from each horizon were collected, avoiding roots, and stored at 20 °C prior to DNA extraction. The remaining sample from each horizon was oven dried at 105 °C prior to soil chemistry analysis.

### Soil property measurements

Soil chemical analysis was conducted on pooled samples from the 0–10 cm horizon for each tree at the University of Delaware Soil Testing Laboratory (Sup. Table 3–4). Total organic C (TOC) and total nitrogen (TN) were measured by high temperature (1200 °C) combustion of 5 g of soil sample using an Elemental Vario Max CN Analyzer. Soil total organic matter (OM) content was measured by loss on ignition (OM-LOI [% weight loss]), and soil pH was measured in a 1:1 soil/distilled water suspension. Plant available nutrients (P, K, Ca, Mg, Mn, Zn, Cu, Fe, and Al) were extracted by Mehlich-III (weak acid solution composed of 0.2 M glacial acetic acid, 0.25 M ammonium nitrate, 0.015 M ammonium fluoride, 0.013 M nitric acid, and 0.001 M ethylene diamine tetraacetic acid (EDTA)) and analyzed by inductively coupled plasma optical emission spectroscopy using a Thermo Iris Intrepid II XSP Duo View ICP.

### Microbial community analysis

Genomic DNA from all tree samples was extracted from the 0–10 cm horizon soil samples using MO BIO Power Soil DNA Kit (Carlsbad, CA, USA) following manufacturer's instructions. DNA was quantified fluorometrically using the Qubit sDNA BR Assay with a Qbit 1.0 fluorometer (Life Technologies, Carlsbad, CA, USA). Sample DNA was analyzed by sequencing the 16S rRNA gene V3-V4 hypervariable region (via V3 kit reagents producing paired-end 325 bp reads) using MiSeq (Illumina, CA [University of Delaware DNA Sequencing & Genotyping Center]). PCR was preformed according to the protocol described by^75^ using the 341F/806R universal primers targeting the V3-V4 hypervariable region:

- 341F(AATGATACGGCGACCACCGAGATCTACACTATGGTAATTGTCCTACGGGAGGCAGCAG;
- 806RCAAGCAGAAGACGGCATACGAGAT**TCCCTTGTCTCC** AGTCAGTCAGCCGGACTACHVGGGTWTCTAAT (bold region represents appropriate barcode sequence),

Sequence data was processed using the Quantitative Insights Into Microbial Ecology 2 (QIIME2) version 2018.2^76^. Reads were filtered using QIIME2 quality filters to remove low-quality, ambiguous and chimeric reads, then trimmed to remove primers and achieve a consistent length. Qualified sequences were denoised using DADA2^77^ and organized into unique amplicon sequence variants (ASVs). The taxonomic identity of each ASV was determined using a custom RDP classifier trained against the amplified region (341–806) of the SILVA SSU database (v132—99% OTUs^78^).

Samples were rarified to 6095 sequences each for downstream diversity analysis to ensure consistent sequencing depth across all samples. Sequences obtained from this study have been deposited in the NCBI Sequence Read Archive (SRA) under BioProject (687277).

### Statistical analysis

Data normality and homogeneity were reviewed for both soil and bacterial relative abundance data prior to analysis of variance (ANOVA). No data transformations were required. One-way ANOVA was used to test for differences in soil chemistry and bacterial relative abundance between trees (beech vs. yellow poplar), forest location (edge vs interior), and forest type (rural, suburban, urban). When F-ratios were significant (p < 0.05), treatment means were compared via Tukey Kramer HSD using SAS-JMP Pro v 14^79^. Additionally, we used linear regression to assess the relationship between the phyla/genera mean relative abundance (%) of each tree (n = 12) and the mean of specific soil chemistry characteristics of all trees sampled (n = 12) using SAS-JMP Pro v 14^78^.

All ordination data analyses were conducted in R (Version 3.3.3^80^). To determine similarities in SMC across forest type, location, and tree species, nonmetric multidimensional scaling (NMDS) analysis was conducted using metaMDS in the vegan package (version 2.5-3^81^). The SØrensen (Bray–Curtis) distance measure and a random starting configuration were used on real data runs. The original data matrix consisted of DNA sequences at each forest type and tree species on the forest edge and in the forest interior. To minimize stress and maximize correlation between variables with ordination configuration, a three-dimensional NMDS ordination solution was selected with satisfactory final stress (0.1771). Correlations between phyla importance values and dimension scores for each site were used to assess phyla contributions to explaining SMC patterns. To assess differences in SMC across forest types (i.e., rural, suburban, urban) and tree species, we used the adonis function in the vegan package to perform permutation multivariate ANOVA to test differences in centroid means and the betadisper function in the vegan package to test multivariate homogeneity of group dispersions. Finally, principal component analysis (PCA) was performed to determine whether patterns in soil chemistry were discernible among forests, trees, and sampling locations using the prcomp function.

## Supplementary Information

Supplementary Information.

## Data Availability

The soil chemistry and sequence datasets generated and analyzed during the current study are available from the corresponding author on reasonable request. Illumina-sequence data [from both beech and yellow poplar soils] has been deposited in the NCBI Sequence Read Archive under BioProject 687277.
