# Supplementary material for: Urbanization pressures alter tree rhizosphere microbiomes
Source: Sci Rep. 2021 May 3;11:9447. doi: 10.1038/s41598-021-88839-8 (PMC8093231; doi:10.1038/s41598-021-88839-8)
Supplement: Supplementary file 1 — Supplementary Information. [file 41598_2021_88839_MOESM1_ESM.docx]

**Urbanization Pressures Alter Tree Rhizosphere Microbiomes**

Carl L. Rosier^1,2^, Shawn W. Polson^3^, Vincent D’ Amico^4^, Jinjun Kan^5^, Tara L.E. Trammell^1^

^1^ Department of Plant and Soil Sciences, University of Delaware Newark, DE 19716

^2^ Rodale Institute Midwest Organic Center, Marion, IA 52302

^3^Center for Bioinformatics and Computational Biology, Delaware Biotechnology Institute, University of Delaware Newark, DE 19713

^4^ US Forest Service, Northern Research Station, Department of Entomology and Wildlife Ecology, University of Delaware, Newark, DE 19716

^5^Stroud Water Research Center, Department of Microbiology, Avondale, PA 19311

*Corresponding author: Carl L. Rosier: crosier@rodaleinst.org,

Ph #: 319-449-1970: ext. 319

Rodale Institute Midwest Organic Center

3706 St, Peters Rd

Marion, Iowa 52302, USA

a)

b)

c)

d)

Figure 1, a) Interior and b) Edge species evenness assessed via 16S rRNA gene sequences isolated from microbial community members at the phylum level (mean + standard error). c) Interior and d) Edge species richness determined by 16S rRNA gene sequences isolated from microbial community members at the phylum level. Samples are separated by forest type: urban (orange) suburban (green) and rural (blue) and tree (column pattern).


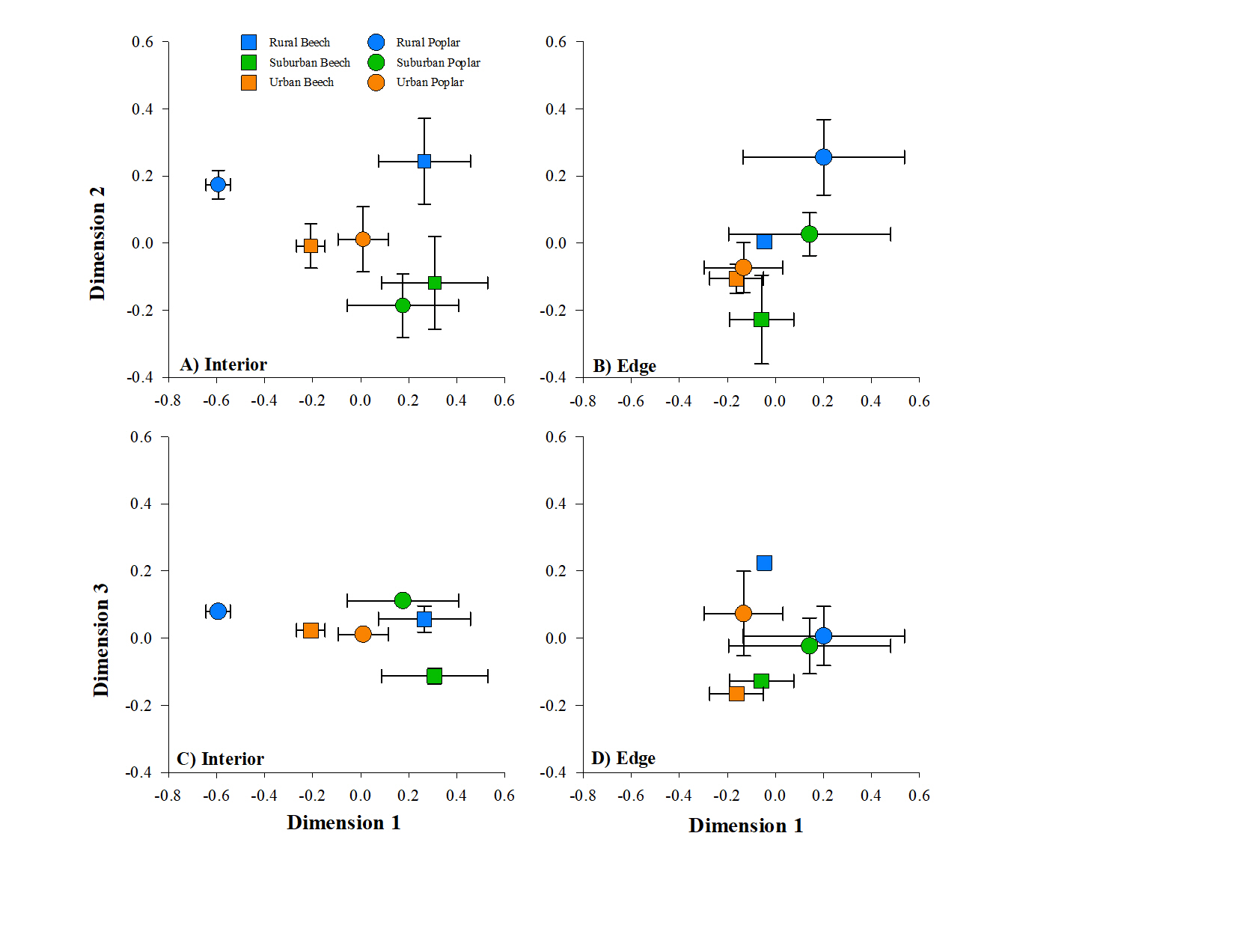


d)

c)

b)

a)

Figure 2. Nonmetric multidimensional scaling (NMDS) ordination comparing beech and yellow poplar microbial community (phyla level composition) in the forest interior (a and c) and along the forest edge (b and d). Urban forest soils are shown in orange, suburban in green, and rural in blue. Each point represents the average of three sampled trees (+ standard error).


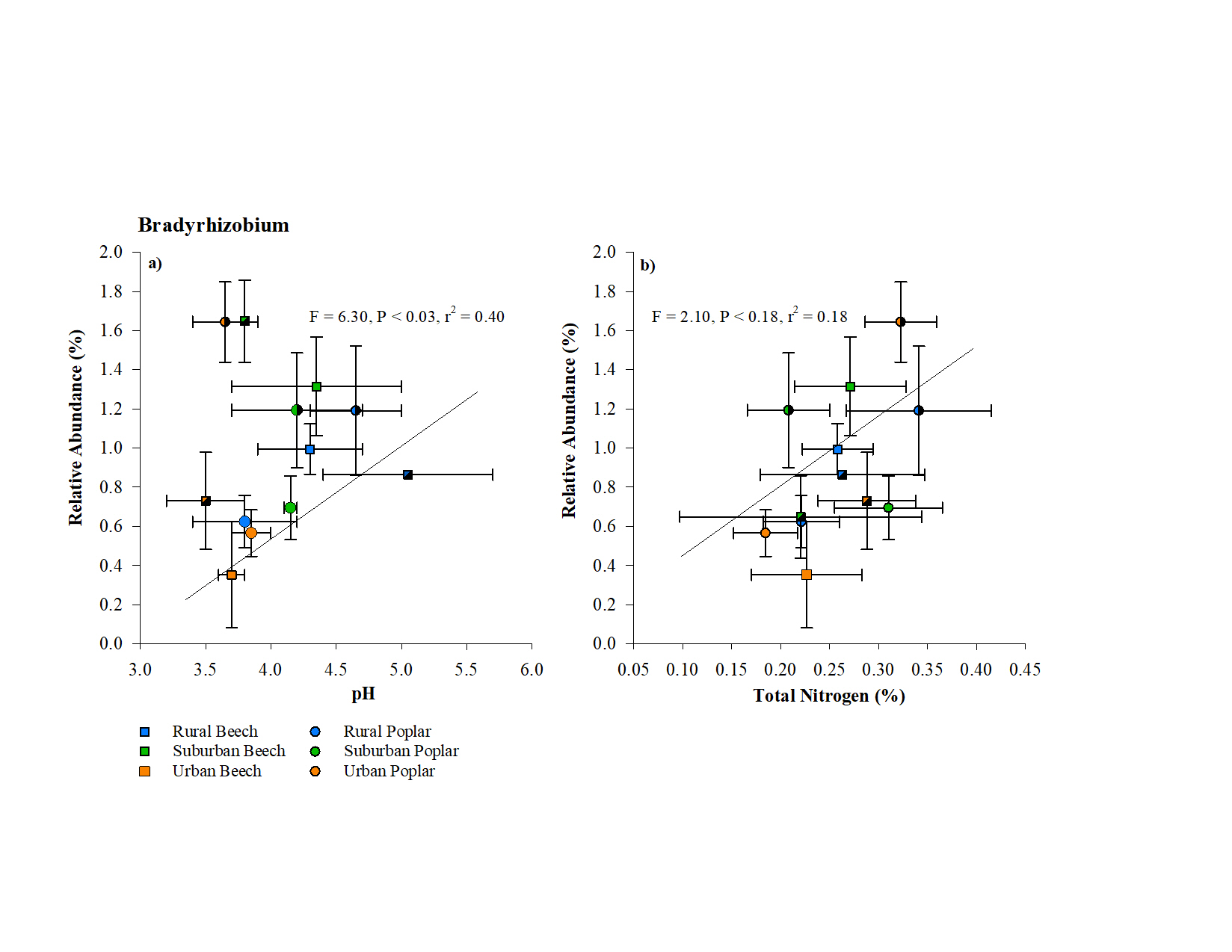


Figure 3. a) Relationship between Bradyrhizobium soil pH and Relative Abundance (%) (b) and the relationship between Bradyrhizobium % N and Relative Abundance (%). Samples are separated by forest type: urban (orange) suburban (green) and rural (blue), tree species (symbol shape), and location (Interior: open symbols vs. Edge: hashed symbols). Each point represents the average of three sampled trees (+ standard error).


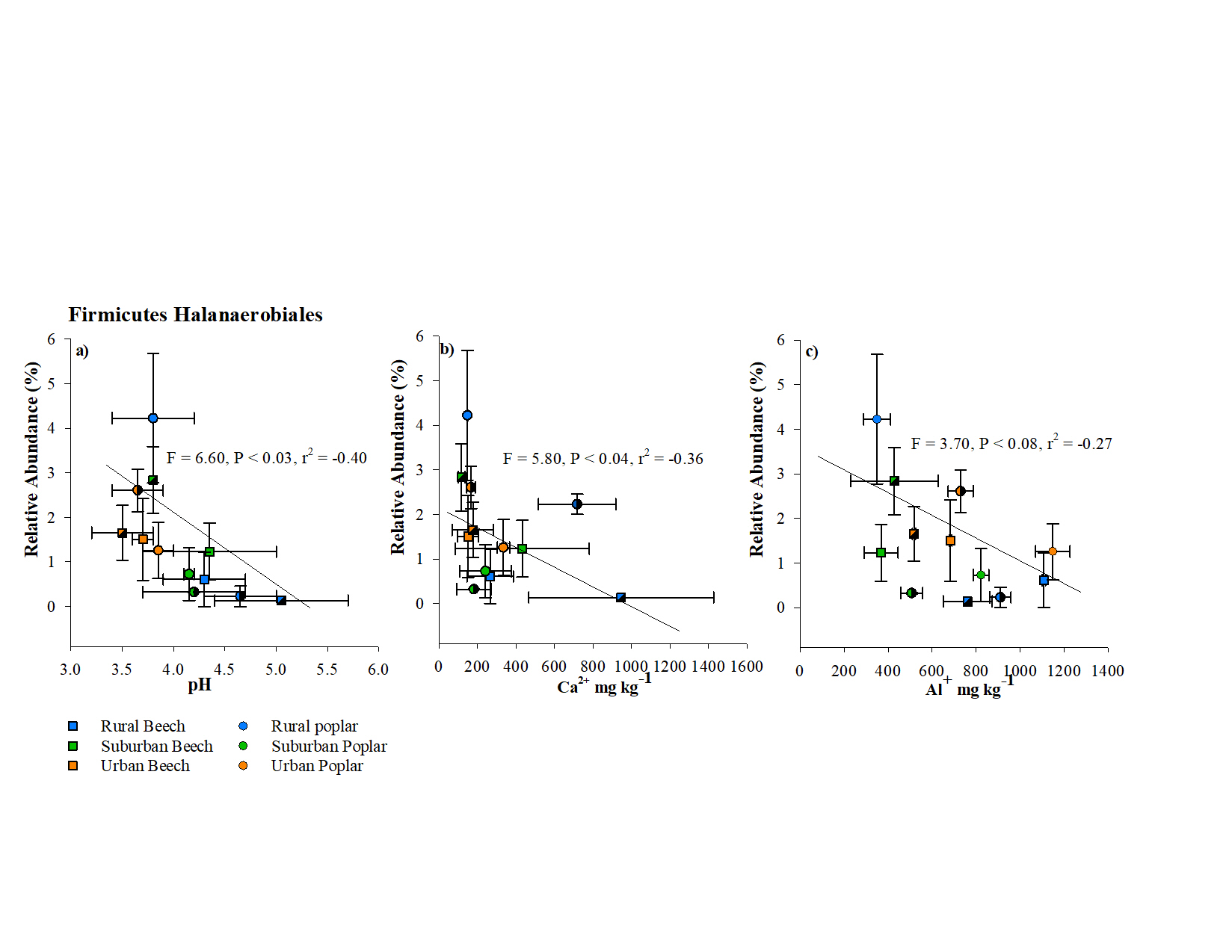


Figure 4. a) Relationship between Firmicutes Halanaerobiales soil pH and Relative Abundance (%), b) represents the relationship between Ca^2+^ (mg kg^-1^) and Firmicutes Halanaerobiales Relative Abundance (%) and c) Al^+^ (mg kg^-1^) and Firmicutes Halanaerobiales Relative Abundance (%). Samples are separated by forest type: urban (orange) suburban (green) and rural (blue), tree species (symbol shape), and location (Interior: open symbols vs. Edge: hashed symbols). Each point represents the average of three sampled trees (+ standard error).


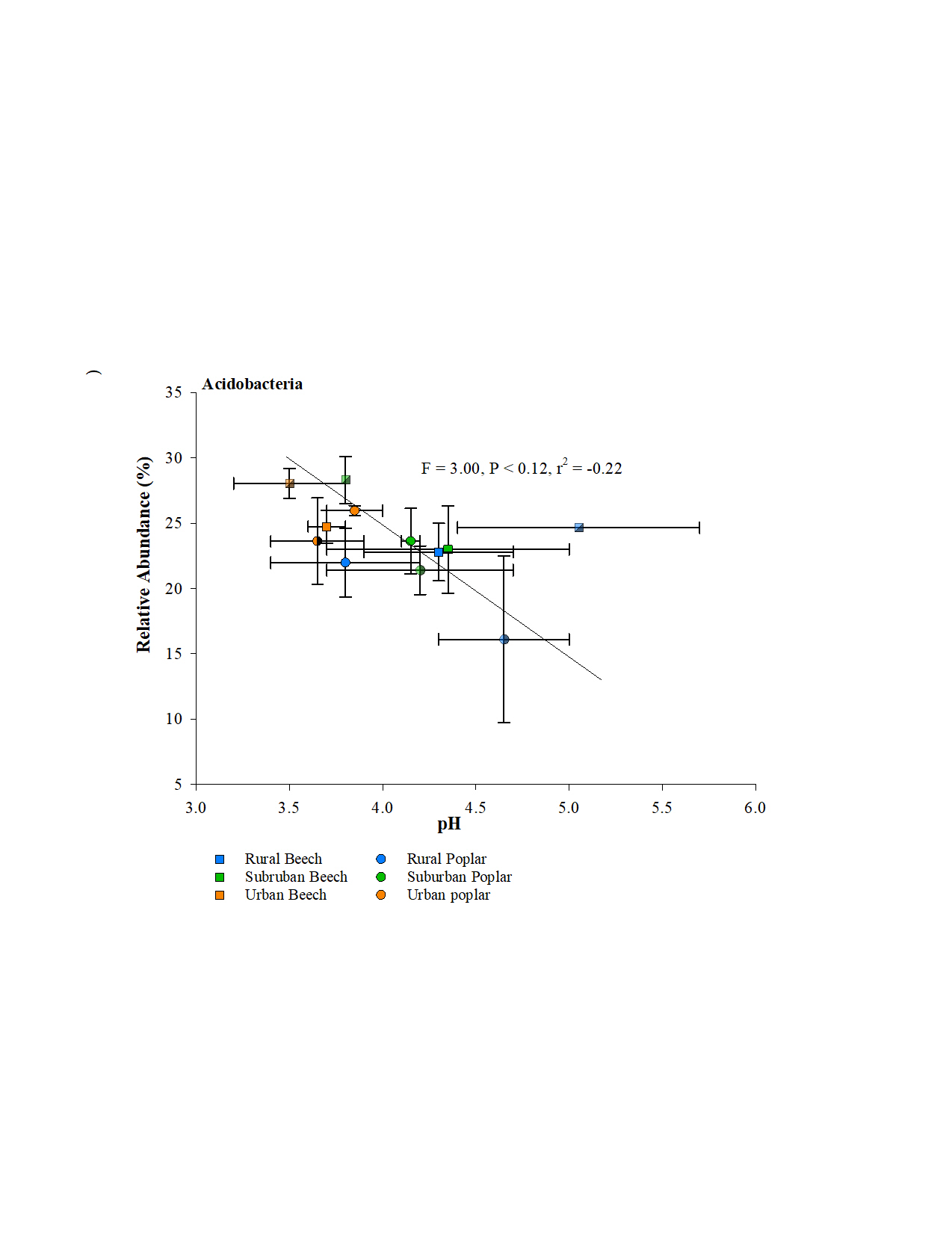


Figure 5) Relationship between soil pH and Acidobacteria Relative Abundance (%). Samples are separated by forest type: urban (orange) suburban (green) and rural (blue), tree species (symbol shape), and location (Interior: open symbols vs. Edge: hashed symbols). Each point represents the average of three sampled trees (+ standard error).


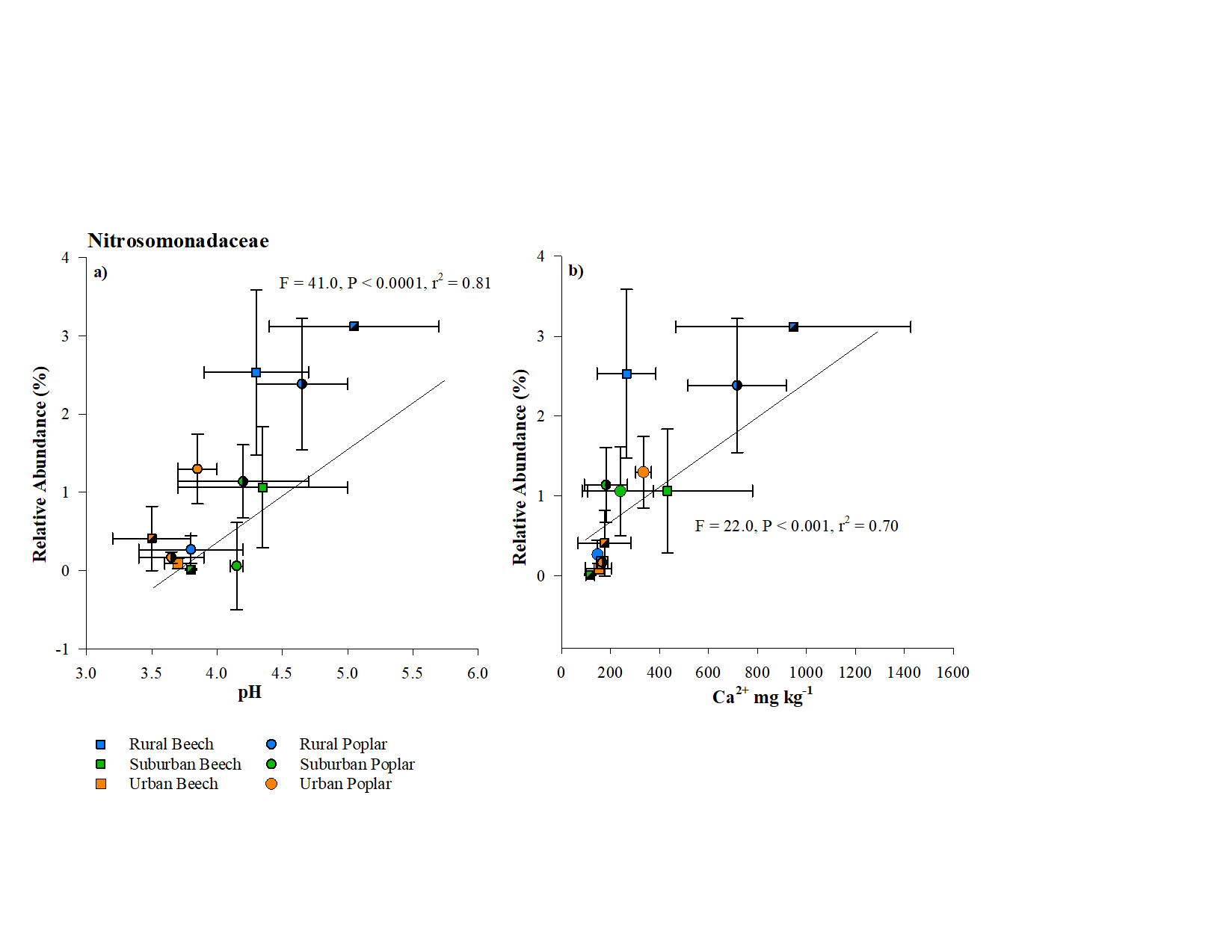


Figure 6, a) Relationship between soil pH and Nitrosomonadaceae Relative Abundance (%), b) is the relationship between Ca^+2^ (mg kg^-1^) and Nitrosomonadaceae Relative Abundance (%). Samples are separated by forest type: urban (orange) suburban (green) and rural (blue), tree species (symbol shape), and location (Interior: open symbols vs. Edge: hashed symbols). Each point represents the average of three sampled trees (+ standard error).


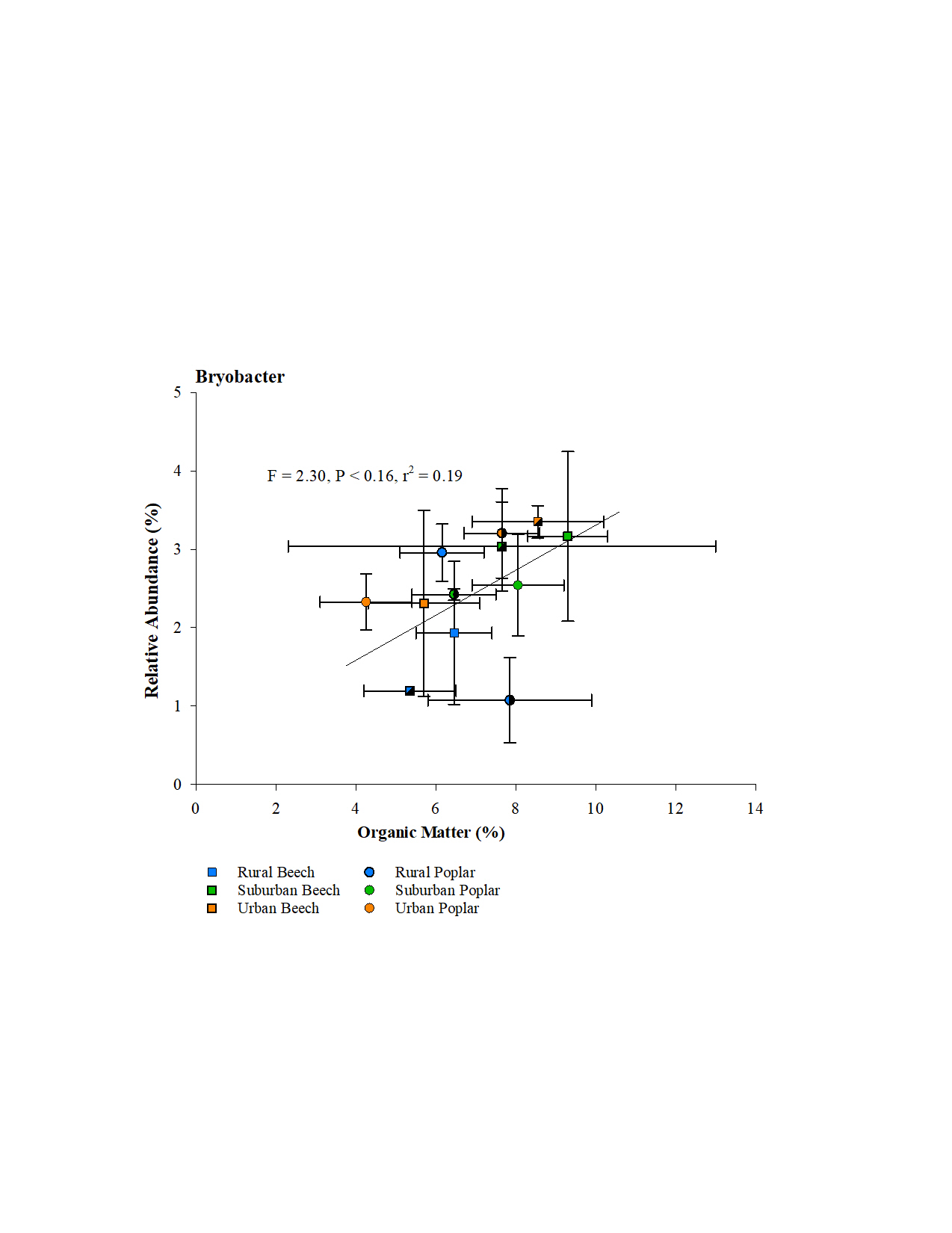


Figure 7) The relationship between Organic matter (%) and Bryobacter Relative Abundance (%).. Samples are separated by forest type: urban (orange) suburban (green) and rural (blue), tree species (symbol shape), and location (Interior: open symbols vs. Edge: hashed symbols). Each point represents the average of three sampled trees (+ standard error).

Tables

**Table: 1 Forest Interior Microbial Community**

|  |  | **Beech** | | |  | **Poplar** | | |  |
| --- | --- | --- | --- | --- | --- | --- | --- | --- | --- |
| **Phylum** | **Genus** | **Rural** | **Suburban** | **Urban** |  | **Rural** | **Suburban** | **Urban** | **F (p)** |
|  | Bradyrhizobium -* | 1.00 (0.13)^ab^ | 1.30 (025)^a^ | 0.35 (0.12)^d^ |  | 0.62 (0.13)^cd^ | 0.70 (0.16)^bc^ | 0.57 (0.12)^cd^ | **3.30 (0.04)** |
| Proteobacteria | Caulobacteraceae *- | 0.13 (0.03)^c^ | 0.15 (0.06)^c^ | 0.18 (0.04)^c^ |  | 0.50 (0.01)^a^ | 0.35 (0.02)^b^ | 0.10 (0.01)^c^ | **3.40 (0.03)** |
|  | Variibacter | 0.25 (0.02) | 1.10 (0.05) | 0.71 (0.38) |  | 0.68 (0.17) | 1.30 (0.18) | 0.51 (0.16) | 2.70 (0.08) |
| Acidobacteria | Blastocatellacea | 0.50 (0.05) | 0.15 (0.01) | 0.00 (0.00) |  | 0.07 (0.01) | 0.05 (0.01) | 0.40 (0.02) | 2.60 (0.08) |
|  | Holophagae | 0.60 (0.10) | 0.30 (0.06) | 0.02 (0.01) |  | 0.13 (0.07) | 0.35 (0.12) | 0.75 (0.20) | 2.70 (0.08) |
| Firmicutes | Halanaerobiales +- | 0.61 (0.10)^c^ | 1.23 (0.30)^b^ | 1.50 (0.45)^b^ |  | 4.20 (0.60)^a^ | 0.73 (0.30)^bc^ | 1.30 (0.63)^bc^ | **3.30 (0.05)** |
| Armatimonadetes | Chthonomonadaceae -* | 0.44 (0.04)^a^ | 0.18 (0.07)^c^ | 0.18 (0.05)^c^ |  | 0.35 (0.06)^ab^ | 0.25 (0.06)^bc^ | 0.51 (0.09)^a^ | **3.60 (0.02)** |
| Planctomycetes | Pirellula | 1.80 (0.30) | 0.58 (0.15) | 0.17 (0.06) |  | 0.41 (0.17) | 0.54 (0.15) | 1.10 (0.20) | 2.50 (0.10) |
|  | Tepidisphaerale -* | 1.10 (0.12)^b^ | 1.00 (0.03)^b^ | 0.43 (0.11)^c^ |  | 1.20 (0.22)^ab^ | 0.41 (0.10)^c^ | 1.50 (0.06)^a^ | **3.70 (0.05)** |

**Table 1.** Significant variations in bacterial genera within the dominate phyla influencing NMDS (Fig 4a and 4c) forest interior soils comparing both beech and yellow poplar. Changes in relative abundance are outlined as + gain, - loss, and * no change as proximity to urbanization increases for beech and poplar trees, respectively. Data presented correspond to percentage (%) mean relative abundance (+ standard error of mean). For each genus, significant differences are presented by different letters comparing forest type (i.e. Rural, Suburban, and Urban) and tree species (beech and yellow poplar): ANOVA, **p < 0.05**.

**Table: 2 Forest Edge Microbial Community**

|  |  | **Beech** | | |  | **Poplar** | | |  |
| --- | --- | --- | --- | --- | --- | --- | --- | --- | --- |
| **Phylum** | **Genus** | **Rural** | **Suburban** | **Urban** |  | **Rural** | **Suburban** | **Urban** | **F (p)** |
|  | Acidibacter ++ | 0.64 (0.00)^c^ | 2.00 (0.17)^a^ | 1.41 (0.15)^b^ |  | 0.70 (0.13)^c^ | 1.00 (0.11)^c^ | 1.40 (012)^b^ | **4.00 (0.03)** |
|  | Burkholderiales | 0.26 (0.00) | 1.70 (0.23) | 0.78 (0.20) |  | 0.26 (0.13) | 0.54 (0.17) | 0.40 (0.02) | 2.80 (0.08) |
|  | Desulfurellales - - | 0.90 (0.00)^b^ | 0.06 (0.02)^c^ | 0.07 (0.02)^c^ |  | 1.40 (0.30)^a^ | 1.10 (0.02)^ab^ | 0.05 (0.01)^c^ | **3.20 (0.05)** |
|  | Nitrosomonadaceae - - | 3.10 (0.00)^a^ | 0.01 (0.001)^e^ | 0.41 (0.02)^d^ |  | 2.40 (0.40)^b^ | 1.13 (0.23)^c^ | 0.17 (0.07)^e^ | **6.00 (0.008)** |
| Proteobacteria | Polyangiaceae + + | 0.00 (0.00)^c^ | 1.00 (0.20)^a^ | 0.54 (0.07)^b^ |  | 0.10 (0.02)^c^ | 0.20 (0.07)^c^ | 0.84 (0.02)^a^ | **7.50 (0.004)** |
|  | Rhodospirillales + + | 0.00 (0.00)^c^ | 1.70 0.10)^a^ | 1.40 (0.20)^a^ |  | 0.20 (0.02)^c^ | 0.20 (0.10)^c^ | 0.90 (0.07)^b^ | **7.20 (0.004)** |
|  | Rhodobiaceae - - | 0.16 (0.00)^b^ | 0.02 (0.001)^c^ | 0.06 (0.001)^c^ |  | 0.35 (0.02)^a^ | 0.12 (0.01)^b^ | 0.06 (0.02)^c^ | **6.00 (0.005)** |
|  | Xanthomonadales | 0.31 (0.00) | 0.22 (0.06) | 0.10 (0.02) |  | 0.53 (0.07) | 0.00 (0.00) | 0.04 (0.01) | 3.10 (0.06) |
|  | Acidimicrobiales - - | 0.28 (0.00)^b^ | 0.01 (0.01)^c^ | 0.16 (0.05)^c^ |  | 0.35 (0.02)^a^ | 0.11 (0.05)^c^ | 0.06 (0.05)^c^ | **10.0 (0.001)** |
|  | Acidobacteriaceae + + | 3.70 (0.00)^b^ | 7.80 (1.25)^a^ | 5.00 (0.90)^b^ |  | 1.40 (0.60)^c^ | 2.30 (0.04)^c^ | 4.10 (0.78)^b^ | **3.50 (0.04)** |
|  | Acidothermus + + | 0.67 (0.00)^d^ | 5.20 (0.70)^a^ | 2.00 (0.39)^b^ |  | 0.54 (0.20)^d^ | 1.10 (0.04)^c^ | 2.50 (0.25)^b^ | **7.30 (0.004)** |
| Acidobacteria | Blastocatellaceae - - | 0.50 (0.00)^a^ | 0.00 (0.00)^b^ | 0.10 (0.05)^b^ |  | 0.54 (0.11)^a^ | 0.76 (0.06)^a^ | 0.10 (0.01)^b^ | **5.50 (0.01)** |
|  | Bryobacter + + | 1.20 (0.00)^c^ | 2.70 (0.70)^ab^ | 3.40 (0.21)^a^ |  | 1.10 (0.50)^c^ | 2.40 (0.07)^b^ | 3.20 (0.60)^a^ | **3.20 (0.05)** |
|  | Edaphobacter | 0.35 (0.00) | 0.57 (0.03) | 0.35 (0.03) |  | 0.23 (0.05) | 0.27 (0.05) | 0.25 (0.05) | 3.00 (0.06) |
|  | Holophagae - - | 0.78 (0.00)^a^ | 0.03 (0.01)^c^ | 0.04 (0.01)^c^ |  | 0.40 (0.05)^b^ | 0.33 (0.02)^b^ | 0.06 (0.03)^c^ | **11.2 (0.001)** |
| Firmicutes | Halanaerobiales + + | 0.03 (0.00)^d^ | 3.00 (0.90)^a^ | 1.70 (0.30)^b^ |  | 0.23 (0.02)^c^ | 0.32 (0.02)^c^ | 2.60 (0.23)^a^ | **3.80 (0.03)** |
|  | Isosphaera + - | 0.20 (0.00)^c^ | 2.10 (0.21)^a^ | 1.50 (0.12)^b^ |  | 0.70 (0.17)^c^ | 1.70 (0.19)^ab^ | 0.44 (0.07)^c^ | **5.50 (0.01)** |
| Planctomycetes | Pirellula | 0.35 (0.00) | 0.10 (0.01) | 0.11 (0.09) |  | 1.20 (0.40) | 0.10 (0.35) | 0.35 (0.03) | 3.10 (0.06) |
|  | Chthoniobacterales | 0.50 (0.00) | 0.10 (0.01) | 0.37 (0.06) |  | 0.40 (0.04) | 0.45 (0.02) | 0.25 0.08) | 3.00 (0.06) |
| Verrucomicrobia | Spartobacteria - + | 0.77 (0.00)^a^ | 0.00 (0.00)^c^ | 0.15 (0.07)^c^ |  | 0.03 (0.01)^c^ | 0.51 (0.09)^a^ | 0.34 (0.06)^b^ | **5.70 (0.01)** |

**Table 2.** Significant variations in bacterial genera within the dominate phyla influencing NMDS (Fig 4b and 4d) forest edge soils comparing both beech and yellow poplar . Changes in relative abundance are outlined as + increase, - decrease, and * no change as proximity to urbanization increases. Data presented correspond to percentage (%) mean relative abundance (+ standard error of mean). For each genus significant differences are presented by different letters comparing forest type (i.e. Rural, Suburban, and Urban) and tree species (beech and yellow poplar): ANOVA, p < 0.05.

Table 3: Summary of Interior Forest Soil Properties within the Proximal Area (< 0.5m) of American beech and yellow poplar trees

|  | **American** | **Beech** |  |  |  | **Yellow** | **Poplar** |  |  |
| --- | --- | --- | --- | --- | --- | --- | --- | --- | --- |
| **Soil Parameters** | **Rural** | **Suburban** | **Urban** |  | **Rural** | **Suburban** | **Urban** | **F (P)** |  |
| pH^a^ | 4.30 (0.40) | 4.40 (0.65) | 3.70 (0.10) |  | 3.90 (0.15) | 3.80 (0.40) | 4.20 (0.05) | 3.50 (0.10) |  |
| OM (%)^b^ | 6.50 (1.30) | 9.30 (1.00) | 5.70 (1.40) |  | 6.10 (1.20) | 8.10 (1.20) | 4.30 (1.60) | 2.60 (0.15) |  |
| TC (%)^c^ | 3.70 (0.30) | 4.30 (0.60) | 3.80 (0.50) |  | 3.40 (0.70) | 4.60 (1.10) | 3.00 (0.50) | 1.50 (0.30) |  |
| TN (%)^c^ | 0.25 (0.05) | 0.27 (0.08) | 0.23 (0.08) |  | 0.22 (0.06) | 0.31 (0.08) | 0.19 (0.05) | 0.87 (0.60) |  |
| C/N | 14.4 (1.20) | 16.4 (2.00) | 17.5 (2.70) |  | 15.5 (0.40) | 14.7 (0.11) | 16.2 (1.10) | 0.57 (0.73) |  |
| Base Sat (%) | 33.0 (4.30) | 51.3 (2.90) | 22.0 (1.2) |  | 20.0 (2.60) | 34.0 (2.0) | 31.0 (1.0) | 9.60 (0.09) |  |
| Al (mg kg^-1^)^e^ | 1110 (8.00) | 368 (78.0) | 684.0 (5.30) |  | 1150 (79.0) | 349 (61.0) | 822 (35.2) | 10.2 (0.07) |  |
| B (mg kg^-1^)^e^ | 1.40 (0.05) | 1.30 (0.70) | 0.80 (0.17) |  | 1.00 (0.06) | 1.00 (0.3) | 1.22 (0.04) | 0.51 (0.76) |  |
| Cu (mg kg^-1^)^e^ | 1.70 (0.04) | 1.30 (0.60) | 1.22 (0.30) |  | 2.20 (0.20) | 0.90 (0.08) | 1.80 (0.16) | 2.70 (0.13) |  |
| K (mg kg^-1^)^e^ | 65.0 (6.20) | 31.4 (6.40) | 53.0 (11.0) |  | 55.0 (1.20) | 25.1 (5.10) | 61.0 (2.20) | 1.90 (0.22) |  |
| Mn (mg kg^-1^)^e^ | 168 (21.0) | 171 (8.40) | 29.1 (5.00) |  | 41.0 (3.00) | 24.1 (5.30) | 61.0 (6.20) | 2.00 (0.21) |  |
| Zn (mg kg^-1^)^e^ | 3.30 (0.91) | 4.30 (0.36) | 2.40 (1.00) |  | 1.30 (0.13) | 1.90 (0.60) | 3.60 (0.75) | 0.62 (0.70) |  |
| Ca (mg kg^-1^)^e^ | 365 (20.0) | 810 (25.0) | 150 (54.0) |  | 146 (2.10) | 340 (32.0) | 334 (32.1) | 8.00 (0.10) |  |
| Fe (mg kg^-1^)^e^ | 175 (25.0)^bc^ | 157 (16.70)^c^ | 426 (18.0)^a^ |  | 312 (13.0)^abc^ | 402 (15.0)^a^ | 377 (13.0)^ab^ | **9.80 (0.01)** |  |
| Mg (mg kg^-1^)^e^ | 70.1 (12.7) | 187 (36.0) | 58.4 (6.50) |  | 44.0 (1.20) | 108 (14.0) | 112 (2.30) | 0.66 (0.67) |  |
| P (mg kg^-1^)^e^ | 42.0 (8.40)^ab^ | 19.0 (2.40)^b^ | 43.0 (4.40)^ab^ |  | 59.0 (2.00)^a^ | 62.0 (1.40)^a^ | 41.0 (3.00)^ab^ | **6.80 (0.02)** |  |

^a^(1:1 v:v) measured in deionized water-Accumet pH meter

^b^(LOI) loss on ignition-Blue-M High Temperature Furnace

^c^measured by combustion using an Elementar Vario Max CN Analyzer

^d^EA-IRMS, CE Instruments NC2500 EA and Thermo-Finnigan Delta Plus XL IRMS

^e^soil extracted using the Mehlich-1and analyzed for plant available nutrients via ICP-OES

|  | **American** | **Beech** |  |  |  | **Yellow** | **Poplar** |  |  |
| --- | --- | --- | --- | --- | --- | --- | --- | --- | --- |
| **Soil Parameters** | **Rural** | **Suburban** | **Urban** |  | **Rural** | **Suburban** | **Urban** | **F (P)** |  |
| pH^a^ | 5.10 (0.70) | 4.20 (0.50) | 3.70 (0.30) |  | 3.80 (0.00) | 3.50 (0.30) | 4.70 (0.40) | 2.40 (0.16) |  |
| OM (%)^b^ | 5.40 (1.20) | 6.50 (1.10) | 7.70 (1.00) |  | 7.60 (1.35) | 8.60 (1.70) | 7.90 (2.10) | 0.20 (0.95) |  |
| TC (%)^c^ | 3.70 (0.51) | 3.40 (1.20) | 5.20 (0.42) |  | 3.80 (1.90) | 5.00 (0.61) | 4.10 (1.00) | 0.48 (0.78) |  |
| TN (%)^c^ | 0.26 (0.08) | 0.21 (0.04) | 0.33 (0.04) |  | 0.22 (0.12) | 0.30 (0.04) | 0.34 (0.07) | 0.53 (0.75) |  |
| C/N | 15.0 (2.80) | 15.7 (2.50) | 16.0 (0.40) |  | 18.0 (1.60) | 17.3 (0.90) | 12.1 (0.40) | 1.50 (0.33) |  |
| Base Sat (%) | 65.2 (10.4) | 24.2 (.10) | 25.1 (1.00) |  | 54.0 (6.00) | 34.2 (0.80) | 15.0 (4.10) | 3.30 (0.09) |  |
| Al (mg kg^-1^)^e^ | 760 (61.3) | 510 (50.0) | 730 (56.0) |  | 430 (51.0) | 517 (8.00) | 910 (47.0) | 3.50 (0.08) |  |
| B (mg kg^-1^)^e^ | 1.40 (0.34) | 1.00 (0.21) | 0.80 (0.30) |  | 1.00 (0.03) | 0.70 (0.10) | 1.60 (0.40) | 1.90 (0.22) |  |
| Cu (mg kg^-1^)^e^ | 2.00 (0.31) | 1.30 (0.50) | 1.20 (0.20) |  | 1.10 (0.20) | 1.10 (0.10) | 2.30 0.06) | 4.30 (0.06) |  |
| K (mg kg^-1^)^e^ | 89.0 (15.0)^ab^ | 38.0 (6.00)^bc^ | 40.0 (2.40)^bc^ |  | 44.0 (7.00)^bc^ | 29.0 (5.50)^c^ | 111 (16.0)^a^ | **11.2 (0.01)** |  |
| Mn (mg kg^-1^)^e^ | 208 (35.0) | 163 (75.0) | 66.0 (25.0) |  | 94.0 (32.0) | 36.0 (14.0) | 240 (60.0) | 0.94 (0.52) |  |
| Zn (mg kg^-1^)^e^ | 4.10 (0.90) | 7.40 (2.10) | 3.20 (0.80) |  | 4.60 (0.02) | 4.80 (1.10) | 7.51 (0.30) | 0.97 (0.50) |  |
| Ca (mg kg^-1^)^e^ | 950 (90.0) | 181 (44.0) | 167 (21.00) |  | 116 (17.0) | 175 (39.0) | 720 (100) | 2.60 (0.14) |  |
| Fe (mg kg^-1^)^e^ | 151 (11.0) | 290 (80.0) | 331 (45.0) |  | 203 (66.0) | 390 (4.20) | 124 (10.0) | 3.80 (0.07) |  |
| Mg (mg kg^-1^)^e^ | 144 (24.0) | 141 (17.0) | 55.2 (14.2) |  | 70.0 (15.4) | 56.3 (11.0) | 103 (25.7) | 2.00 (0.22) |  |
| P (mg kg^-1^)^e^ | 56.5 (14.0) | 26.3 (10.0) | 64.0 (5.80) |  | 31.0 (0.31) | 56.0 (5.20) | 60.0 (27.0) | 1.50 (0.33) |  |

Table 4: Summary of Edger Forest Soil Properties within the Proximal Area (< 0.5m) of American beech and yellow Poplar trees

^a^

(1:1 v:v) measured in deionized water-Accumet pH meter

^b^(LOI) loss on ignition-Blue-M High Temperature Furnace

^c^measured by combustion using an Elementar Vario Max CN Analyzer

^d^EA-IRMS, CE Instruments NC2500 EA and Thermo-Finnigan Delta Plus XL IRMS

^e^soil extracted using the Mehlich-1and analyzed for plant available nutrients via ICP-OE
